# Interaction with the host: the role of fibronectin and extracellular matrix proteins in the adhesion of Gram-negative bacteria

**DOI:** 10.1007/s00430-019-00644-3

**Published:** 2019-11-29

**Authors:** Diana J. Vaca, Arno Thibau, Monika Schütz, Peter Kraiczy, Lotta Happonen, Johan Malmström, Volkhard A. J. Kempf

**Affiliations:** 1 Institute for Medical Microbiology and Infection Control, University Hospital, Goethe University Frankfurt am Main, Paul-Ehrlich-Str. 40, 60596 Frankfurt, Germany; 2 Institute for Medical Microbiology and Infection Control, University Hospital, Eberhard Karls-University, Tübingen, Germany; 3 Division of Infection Medicine, Department of Clinical Sciences, Faculty of Medicine, Lund University, Lund, Sweden

**Keywords:** Gram-negative bacteria, Adhesins, Extracellular matrix proteins, Fibronectin, Collagen, Laminin

## Abstract

The capacity of pathogenic microorganisms to adhere to host cells and avoid clearance by the host immune system is the initial and most decisive step leading to infections. Bacteria have developed different strategies to attach to diverse host surface structures. One important strategy is the adhesion to extracellular matrix (ECM) proteins (e.g., collagen, fibronectin, laminin) that are highly abundant in connective tissue and basement membranes. Gram-negative bacteria express variable outer membrane proteins (adhesins) to attach to the host and to initiate the process of infection. Understanding the underlying molecular mechanisms of bacterial adhesion is a prerequisite for targeting this interaction by “anti-ligands” to prevent colonization or infection of the host. Future development of such “anti-ligands” (specifically interfering with bacteria-host matrix interactions) might result in the development of a new class of anti-infective drugs for the therapy of infections caused by multidrug-resistant Gram-negative bacteria. This review summarizes our current knowledge about the manifold interactions of adhesins expressed by Gram-negative bacteria with ECM proteins and the use of this information for the generation of novel therapeutic antivirulence strategies.

## Introduction

The capacity to adhere to host cells and thereby avoid clearance by the host defense systems (e.g., via peristalsis, fluid flow or innate immunity) is an important determinant for a successful colonization by bacterial pathogens. Adhesion to the host cells can facilitate translocation of pathogenic bacteria across the cellular and tissue barriers by generating a stable starting point on which the microorganism can persist, replicate, and internalize into host cellular compartments. Once a stable adhesion to host cells is established, pathogens are able to spread within the host and express and/or release further virulence factors enabling subsequent steps of infections. Such virulence factors include, e.g., bacterial toxins (modulating host cell functions), cell surface carbohydrates or proteins (protecting the bacterium from host defense), and exoenzymes (contributing to bacterial dissemination).

A group of proteins exposed on the pathogen’s surface called “adhesins” has been identified as the molecular basis for bacterial adherence to certain host molecules. The way that different bacterial populations take advantage of their adhesins and how they bind to their specific receptors within the host is decisive for the particular type of disease caused by a particular organism. Adhesins are involved in biofilm formation and have proven to undermine host strategies for pathogen clearance [1, 2]. Furthermore, bacterial adhesion to host cell surfaces activates both bacterial and host signaling, subsequently enabling bacterial spread and the evasion of innate and cellular immune responses [3]. On the host side, the extracellular matrix (ECM) is one of the most important proteinaceous tissue components, due to the wide distribution of ECM in the connective tissue and basement membranes [4]. Targeting of ECM proteins for adherence is, therefore, one of the major strategies for pathogen colonization and host invasion [4].

Bacterial binding capacity to ECM proteins was first described over 40 years ago, with the report of *Staphylococcus aureus* binding to fibronectin [5]. Since then, our knowledge about the mechanisms underlying host–pathogen interactions has increased significantly. This resulted in promising ideas for inhibiting such interactions for the future development of anti-bacterial therapeutics. In this review, we summarize the principal ECM proteins involved in the adhesion processes of Gram-negative bacteria, the impact on virulence and pathogenesis, and how to use this knowledge in terms of generating novel antivirulence-therapeutic strategies.

## Extracellular matrix proteins involved in the adhesion of Gram-negative bacteria

The ECM is a highly dynamic structure having various functions. It consists of numerous macromolecules in charge of, e.g., the structural support and scaffolding of cellular barriers, cellular signaling, and the regulation of physiological processes. The ECM is composed of proteoglycans and glycoproteins secreted locally and brought together into an organized network. The main fibrous proteins forming parts of the ECM are collagen, elastin, fibronectin, laminin, and vitronectin [6], making these molecules a preferred target for bacterial adhesion.

### Collagen

Collagen is the major glycoprotein representing 30% of the total protein content in the human body. Its presence is crucial for maintaining tissue structure, cell adhesion, embryonic development, and many other functions. Apart from mammals and some other vertebrates, collagen has been identified in many invertebrate organisms, evidencing the conservation and importance of the molecule throughout evolution [7, 8]. The latest report described a total of 28 collagen types encoded by more than 45 genes distributed in body tissue and organs [9, 10]. Initially, it was thought that all types of collagen were secreted by fibroblasts which are present in the connective tissue [11] but the production of certain types of collagen by epithelial cells indicates the broad distribution of the molecule in the human body [10]. Under normal conditions, collagen is degraded extracellularly by tissue collagenases, belonging to the class of matrix metalloproteinases [9].

Collagen consists of α-chains and the variability in the number of α-chains present in the molecule defines the different collagen types distributed in the human body. Despite the presence of multiple isoforms and tissue expression levels, all the different types of collagen share common structures [10]. The most significant structure is the presence of Gly-X-Y repeats located in the central part of the α-chain, known as the “collagenous domain”. A triple helix structure is formed by regular hydrogen bonding between proline and glycine residues [12]. In addition to the collagenous domain, there are regions lacking the Gly-X-Y repeats named “non-collagenous domains”. The presence of these long non-collagenous domains along the molecule creates breaks in the triple helix conformation, while the non-collagenous domains in the N-terminal and C-terminal ends are removed by procollagen N- and C-proteinases to allow the assembly into fibrils [13]. The supramolecular association occurs after extracellular release and further assembly into networks or fibrils including other ECM proteins.

The collagen protein family is widely present in skin (collagen type I in association with collagen types III, V, VII, XII, XIII and XIV), in bones (collagen type I in association with collagen types XXIV), in cartilage (collagen type II in association with IX, X, XI and XIII), and in basement membranes (collagen type IV in association with collagen type XVIII) [9, 10]. The presence of collagen-binding proteins (collagen-BPs) in pathogenic bacteria is, therefore, not incidental but has evolved because of the broad distribution of this ECM protein in organs and tissue. The majority of adhesin–host protein interactions observed in Gram-negative bacteria have been associated with collagen type I, IV, and V [4].

### Fibronectin

Fibronectin (Fn) is a multidomain glycoprotein present in body fluids and on cell surfaces with the principal function of connecting the cell to the exterior ECM. Two major forms of Fn are present in the body: a soluble (plasma) and an insoluble (cellular) form. Plasmatic Fn is produced by hepatocytes and is, therefore, present in blood, saliva, and other fluids, playing important roles in blood clotting [14]. Cellular Fn is secreted by fibroblasts and endothelial cells and is incorporated on the cell surface into a fibrillar-type matrix [15, 16]. Turnover of ECM proteins is an important mechanism to remove biologically active proteins from the extracellular environment. Fn degradation occurs intracellularly after endocytosis of non-polymerized Fn molecules [17].

**Fig. 1:**
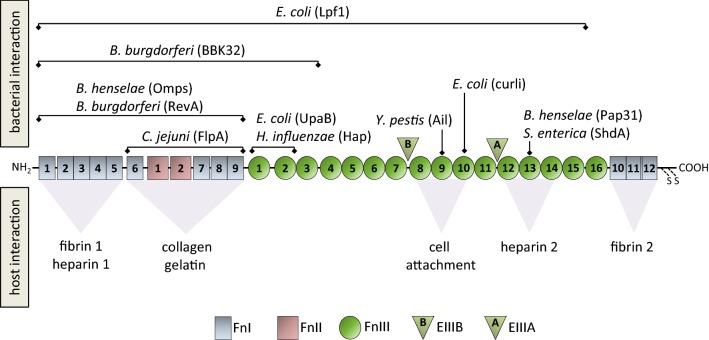
Schematic drawing of the fibronectin molecule (monomer) with selected bacterial protein-binding sites and selected host protein–protein interaction sites. Fibronectin (Fn) is a heterodimer composed of two splice variants connected by a C-terminal disulfide bond. The molecule contains nine Fn type I repeats, two Fn type II repeats, and between 15 and 18 Fn type III repeats. In cellular Fn, EIIIA (A) and EIIIB (B) domains are present as a result of alternative splicing Adapted from [202]

The Fn molecule is a heterodimer composed of two splice variants of about 230 and 270 kDa connected by a C-terminal disulfide bond (see Fig. 1). In general, the Fn structure is organized into 12 type I repeats (FnI), two type II repeats (FnII), and a variable number (between 15 and 18) of type III repeats (FnIII). Differences between the splice variants modify the number of modules in FnIII [15, 16, 18]. Additionally, cellular Fn can include EIIIA and EIIIB domains, which notably are not present in the soluble molecule [19, 20].

Fn mediates important human protein–protein and protein–oligosaccharide interactions during the formation of the ECM [21]. The FnI_1_–FnI_5_ components are the most conserved Fn region across vertebrates [21]. This domain is required for the proper assembly of the ECM and binds to heparin (lower affinity) and fibrin (stronger affinity). Moreover, this domain is also the major fibrin-binding site in the Fn molecule. The interaction between Fn and fibrin is important for cell adhesion, cell migration into fibrin clots, and for macrophage removal from circulation after a trauma or in the case of inflammation. Another region consisting of FnI_6_, FnII_1–2_, and FnI_7–9_, promotes collagen binding. This interaction has been suggested to occur either to mediate cell adhesion or to favor clearance of denatured collagenous material from blood and tissue. The FnIII domain mediates cell attachment via integrins (cell-surface heterodimeric receptors) in the RGD loop located at the FnIII_8–10_ area. The interaction via integrins allows the linkage of ECM with the intracellular cytoskeleton. The FnIII_12–14_ modules contain the strongest interaction site necessary for heparin-binding. It has been proposed that this region facilitates the formation of protein interactions for insoluble fibril assembly, whereas in some cell types the heparin-binding domain promotes cell adhesion. A second fibrin-binding site is located at the C-terminal FnI_10–12_ modules [18, 21–23].

The presence of bacterial Fn-binding proteins (FnBPs) was demonstrated by the inactivation of the respective FnBP genes and the observation of diminished or abolished bacterial adhesive characteristics in mutants lacking the expression of the protein. The observation of a Fn-binding repeat sequence within the adhesins (GGXXXXV(E/D)(F/I)XX(D/E)T(Xx15) EDT) has been described for certain bacterial proteins [24]. On the other hand, a canonical binding site in the Fn molecule located in the FnI_2_–FnI_5_ region has been identified due to the interaction of many FnBPs in this area [22, 25]. Notwithstanding, other non-canonical bacterial binding sites associated with positions FnI_6_, FnII_1–2_, FnI_7–9_, FnIII_9–10_, and FnIII_12_ have also been identified in the Fn molecule (see Fig. 1).

### Laminin

Laminin (Ln) is a multifunctional molecule with a total of 15 heterotrimeric isoforms differentially distributed in basement membranes, connective tissue, cell surface, skin, and blood vessels. This ECM protein is in charge of maintaining the structural scaffold, cell migration, and signaling [4]. Plasmin degrades Ln that is located in the basement membrane at the dermal–epidermal junction and in the hippocampus [26, 27].

**Fig. 2:**
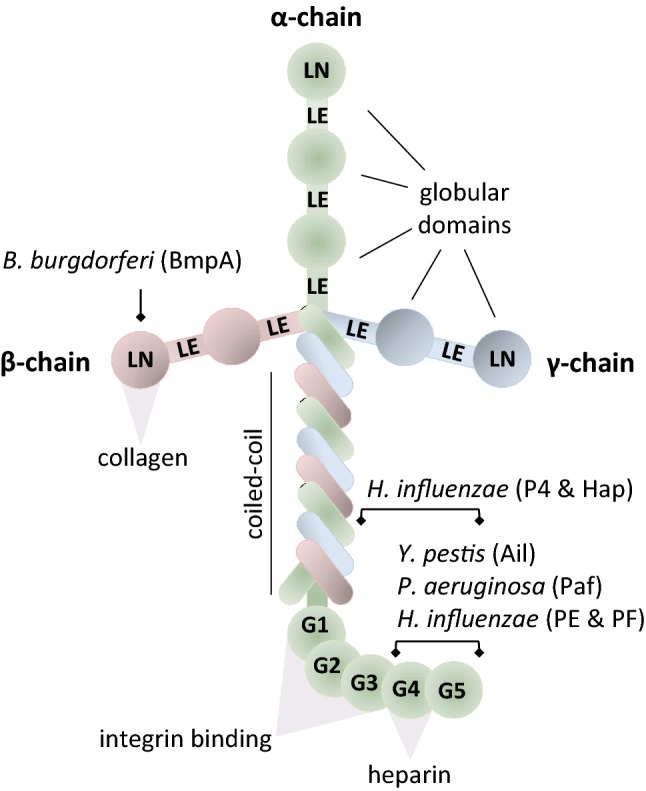
Schematic drawing of the laminin molecule (heterotrimer) with selected bacterial protein-binding sites and selected host protein–protein interaction sites. Laminin (Ln) consists of α- (400 kDa), β- (200 kDa), and γ- (200 kDa) chains interconnected with disulfide bonds. Ln domains (N-terminus) are involved in the polymerization of the molecule. The epidermal growth factor-like (LE) domains interconnect the globular domains. The coiled-coil region is present in all chains and their interactions maintain the structure. The C-terminus contains five globular domains (G1–G5) important for cellular scaffold functions

Ln consists of α-(400 kDa), β-(200 kDa), and γ-(200 kDa) chains, independently expressed and interconnected via disulfide bonds at their C-terminal regions (see Fig. 2). Ln trimerizes prior to its extracellular secretion and then forms networks cooperating with other ECM proteins. The first N-terminus (240–250 amino acid residues: LN domains) are well conserved among Ln isoforms and are involved in the polymerization of the molecule. The epidermal growth factor-like (LE) domains are associated with functions such as signaling, growth, and development and interconnect the globular domains of the Ln molecule. Finally, there is the coiled-coil region that consists of 600 amino acids and interacts with other proteins and receptors [4].

The Ln molecule has been found widely distributed in the renal parenchyma including glomeruli and tubules, and in gastric mucosa. The presence of Ln-Binding Proteins (LnBPs) has been demonstrated in Gram-negative pathogens such as *Escherichia coli*, *Haemophilus influenzae*, *Neisseria meningitidis*, *Helicobacter pylori*, *Yersinia enterocolitica,* and *Borrelia burgdorferi* [4] (see Fig. 2).

## Adhesins of Gram-negative bacteria and cellular matrix protein interactions

Bacterial adherence to host tissues represents the first and decisive step in the infection process. Although bacterial attachment seems to be beneficial for microorganisms, it may become a double-edged sword if, after attachment, the host immune signaling is activated and impedes internalization and phagocytosis strategies. To overcome this problem, bacteria can express surface structures to protect themselves from immune recognition and might employ further traits, such as protein secretion systems to modulate and to evade the host´s immune system. Actually, tight adhesion can be an essential prerequisite to engage these important facilitators of infection [28]. A group of proteins, called adhesins, are in charge of keeping the pathogen in close contact with the host.

Adhesins are a highly diverse group of proteins with heterologous architecture and domain composition [29]. The complexity of the bacterial tools used for cell adhesion ranges from single monomeric proteins to intricate multimeric macromolecules. Among this group, Trimeric Autotransporter Adhesins (TAAs) are a type of adhesins presented on the outer membrane of many human pathogenic Gram-negative bacteria. These obligate homotrimeric proteins are secreted via the type Vc pathway and led across the inner membrane Sec-dependently by an N-terminal signal peptide. The C-terminal β-barrel domain interacts with the β-barrel assembly machinery (BAM) in order to be inserted into the outer membrane [30–32]. Most likely, concurrently with the β-barrel insertion facilitated by the BAM, the long passenger domain (N-terminal: head, neck and stalk domains) is translocated through the barrel in a hairpin-conformation and ends up exposed on the bacterial surface [33, 34]. So far, all the described TAAs have been functionally associated with adhesion properties [30, 35].

With a similar secretion mechanism, the classical autotransporters (“monomeric autotransporter adhesins”) are secreted via the type Va pathway. Briefly, the autotransporter crosses the inner membrane by the Sec machinery and, once the protein is in the periplasm, distinct chaperone proteins maintain the unfolded structure of the autotransporter [30]. BAM recognizes the C-terminal membrane anchor; this step aids the insertion of the β-barrel membrane anchor into the outer membrane [34]. The linker region forms a hairpin inside the barrel while the passenger domain crosses the pore. For some autotransporters, the linker region will be cleaved releasing the passenger domain into the extracellular environment [30, 31]. Monomeric autotransporters [e.g., *Haemophilus* adhesion and penetration protein (Hap), MisL and ShdA of *Salmonella enterica* serotype Typhimurium and UpaB of uropathogenic *E. coli*; see Table 1] are the most ubiquitous class of secreted proteins in Gram-negative bacteria and accomplish varied functions including cell adhesion, biofilm formation, and resistance to host defenses [36].

**Table 1:** Adhesins of Gram-negative bacteria and their interactions with ECM proteins (examples)

Genus	Species	Adhesin	Estimated molecular weight of monomers	Protein data bank code	Class of bacterial adhesin	Binding to ECM^a^	Specific binding site in adhesin	Specific binding site in ECM	Estimated Kd value^b^	References
*Acinetobacter spp.*	*A. baumannii*	Ata	189 kDa	NA	TAA	Collagen I, III, IV, V	Passenger domain four-SVAIG motifs (putative)	Not determined	NA	[40, 41]
						Ln	Not determined	Not determined	NA	[40]
		OmpA (Omp38)	38 kDa	3TD3 3TD4 3TD5 4G4Y 4G4Z 4G88	Porin	Fn	Not determined	Not determined	NA	[42]
		Omp33 (Omp 33-36)	31 kDa	6GIE	Porin	Fn	Not determined	Not determined	NA	[42, 43]
*Bartonella spp.*	*B. henselae*	BadA	327 kDa	3D9X	TAA	Fn	Stalk domain	Not determined	NA	[44, 45]
						Collagen I, III, IV	Head and stalk domain^c^	Not determined	NA	
						Ln	Not determined	Not determined	NA	
		Pap31	31 kDa	NA	Porin	Fn	Not determined	Fn III_13_	NA	[46]
		Omp43	43 kDa	NA	Porin	Fn	Not determined	Heparin and gelatin-binding domains of Fn	NA	[47]
		Omp89	89 kDa	NA	Porin	Fn	Not determined		NA	
	*B. quintana*	VompA	101 kDa	NA	TAA	Collagen IV	Not determined	Not determined	NA	[48]
		VompC	104 kDa	NA						
	*B. bacilliformis*	Brp (Bbad)	~130 kDa	NA	TAA	Fn and collagen binding (putative)	Not determined	Not determined	NA	[49]
		Hbps	Not determined	NA	Hemin-binding proteins	Fn binding (putative)	Not determined	Not determined	NA	
*Borrelia* *spp.*	*B. burgdorferi*	BBK32	47 kDa	6N1L 4PZ5	Surface-exposed lipoprotein	Fn	N-terminal domain	70 kDa N-terminus, gelatin/collagen binding domain, Fn III_1–3_	10 nM (ELISA)	[50, 51]
		BBA33	17 kDa	NA	Surface-exposed lipoprotein	Collagen IV, VI	Not determined	Not determined	collagen VI 350 nM (ELISA)	[52]
		BmpA	39 kDa	NA	Surface-exposed lipoprotein	Ln	Not determined	Collagen binding site (80 aa to C-terminus)	0.1 µM (ELISA)	[53]
		BmpB	37.5 kDa	NA	Surface-exposed lipoprotein	Ln	Not determined	Not determined	NA	[53]
		BmpC	40 kDa	NA	Surface-exposed lipoprotein	Ln	Not determined	Not determined	NA	[53]
		BmpD	37 kDa	NA	Surface-exposed lipoprotein	Ln	Not determined	Not determined	NA	[53]
		CspA (CRASP-1, BbCRASP-1, BBA68, ZS7.A68, FHBP)	25.9 kDa	4BL4	Surface-exposed lipoprotein	Collagen I, III, IV	Not determined	Not determined	NA	[54]
						Fn	Not determined	Not determined	NA	
						Ln	Not determined	Not determined	NA	
		CspZ (CRASP-2, BbCRASP-2, BBH06)	23.2 kDa	6ATG 4BG0 4CBE	Surface-exposed lipoprotein	Fn	Not determined	Not determined	NA	
						Ln	Not determined	Not determined	NA	
		ErpX	40 kDa	NA	Surface-exposed lipoprotein	Ln	Region between N-terminus (30 aa) and C-terminus (31 aa)	Not determined	NA	[55, 56]
		RevA	17 kDa	NA	Surface-exposed protein	Fn	60 aa at the N-terminus	70 kDa N-terminus not inhibited by heparin	12.5 nM (ELISA)	[55, 57]
		RevB	17.5 kDa	NA	Surface-exposed protein	Fn	Not determined	Not determined	NA	[55, 58]
*Campylobacter spp.*	*C. jejuni*	CadF	37 kDa	NA	Porin	Fn	Residues 134-137	Not determined	NA	[59, 60]
		FlpA	46 kDa	NA	Surface-exposed lipoprotein	Fn	FNIII-like repeat D2 (residues 150–164)	Fn gelatin-binding domain	28.7 nM (ELISA)	[61, 62]
		Cj1349c	51 kDa	NA	Surface-exposed lipoprotein	Putative Fn and fibrinogen	Not determined	Not determined	NA	[63]
*Escherichia* *spp.*	Enteropathogenic *E. coli*	Flagellin	50 kDa	NA	Flagella	Fn	YDVGGDAYTVNVDS (putative)	Not determined	NA	[64, 65]
						Collagen	Not determined	Not determined	NA	
						Ln	Not determined	Not determined	NA	
	Enteroaggregative *E. coli*	AaF II	Not determined	4OR1	Fimbriae	Collagen	Major subunit of AAF/II fimbria	Not determined	NA	[66]
						Fn		Not determined	NA	
						Ln		Not determined	NA	
	*E. coli* (several strains)	Curli	15 kDa	6G8C 6G8D 6G8E 6G9G	Curli	Fn	NNS24 and VDQ26 peptides	RGD Motif	NA	[67, 68]
						Ln	Not determined	Not determined	NA	[67, 68]
	Enterohemorrhagic *E. coli*	Lpf1	18 kDa	NA	Fimbriae	Collagen IV	Not determined	Not determined	NA	[69]
						Fn	Not determined	30-, 45-, 70-, and 120-kDa proteolytic fragments	NA	
						Ln	Not determined	Not determined	NA	
	Uropathogenic *E. coli*	UpaB	80 kDa	6BEA	Autotransporter	Fn	Residues D116, D119, N146, N175, D217, K245, D246, D281, R310 and D336	Fn III_1–2_ (putative)	45.2 µM (surface plasmon resonance)	[70, 71]
						Ln	Not determined	Not determined	NA	[71]
*Haemophilus spp.*	*H. influenzae* (NTHi)	Hap	155 kDa	3SYJ	Autotransporter	Fn	511 residues (526–1036) of C-terminal passenger domain	FnIII_1–2_	15 nM (ELISA)	[72–76]
						Collagen IV	Same as above	Not determined	20 nM (ELISA)	
						Ln	Same as above	Heparin-binding site(s) mainly G-like domains 1-5	35 nM (ELISA)	
		PE	18 kDa	3ZH5 3ZH6 3ZH7	Surface-exposed protein	Ln	N-terminus (residues 41-68)	Heparin-binding site(s) mainly G-like domains 4 and 5	1.5 μM (surface plasmon resonance)	[76–79]
		PF	30 kDa	NA	Surface-exposed protein	Ln	N-terminus (residues 23-48)	Heparin-binding site(s) mainly G-like domains 4 and 5	NA	[76]
		P4	28 kDa	3OCU 3OCV 3OCW 3OCX 3OCY 3OCZ 3SF0	Surface-exposed protein	Fn	Core/α-helix domains	Not determined	10.2 nM (ELISA)	[76, 80]
						Ln	Same as above	Heparin-binding site(s) mainly G-like domains 1-5	9.3 nM (ELISA)	
*Helicobacter spp*	*H. pylori*	AlpA	56 kDa	NA	Surface-exposed protein	Ln	Not determined	Not determined	NA	[81]
		AlpB	55 kDa	NA	Surface-exposed protein	Ln	Not determined	Not determined	NA	
*Pseudomonas spp.*	*P. aeruginosa*	OprQ	46 kDa	NA	Porin	Fn	Not determined	Not determined	NA	[82]
		Paf	34 kDa	NA	Surface-exposed protein	Ln	Not determined	Heparin-binding site(s) mainly G-like domains 4 and 5	NA	[83]
*Salmonella spp.*	*S. enterica*	ShdA	200 kDa	NA	Autotransporter	Fn	Residues 470-1553 (passenger domain)	FnIII_13_	0.12 µM (ELISA)	[84–86]
		MisL	101 kDa	NA	Autotransporter	Fn	Non-conserved region passenger domain	Not determined	0.17 µM (ELISA)	[87]
	*S. typhimurium*	Rck	17 kDa	NA	Surface-exposed protein	Fn	Not determined	Not determined	NA	[88]
						Ln	Not determined	Not determined	NA	
		PagC	20 kDa	NA	Surface-exposed protein	Ln	Not determined	Not determined	NA	[88]
*Yersinia spp.*	*Y. enterocolitica*	YadA	47 kDa	1P9H 3LT6 3LT7 2LME	TAA	Collagen	Trimeric form of YadA head domain	Not specific collagen binding sequence	0.28 µM (surface plasmon resonance)	[89, 90]
						Fn	Not determined	Not determined	NA	
						Ln	Not determined	Not determined	NA	
	*Y. pestis*	Ail	17 kDa	5VJ8 2N2L 2N2 M 3QRA 3QRC	Surface-exposed protein	Ln	Not determined	G-like domains 4 and 5	NA	[91]
						Fn	Not determined	Fn III_9_	290 nM (ELISA)	[92, 93]

^a^When defined the particular type of collagen (collagen I–VI) was included

^b^Dissociation constant for adhesin-ECM protein (concentration of ligand required for half-maximal binding activity)

^c^Other domains might be involved

Other types of adhesins described in Gram-negative bacteria are pili or fimbriae. These filamentous surface proteins comprise a scaffold-like domain anchored to the bacterial membrane with strong binding specificities. Interestingly, some pathogens (e.g., *Bartonella bacilliformis*) harbor both types of adhesins (TAA BrpA/BbadA [37] and flagellin [38]). This suggests that such adhesins might play important roles in different conditions or at distinct stages of the infection process. This diversity and variability of adhesins even between species from the same genus of bacteria make the task of deciphering the precise mode of bacteria–host interaction challenging [39]. In the following paragraphs, known ECM binding adhesins will be discussed in more detail in accordance with the Gram-negative pathogen they belong (see Table 1).

### *Acinetobacter baumannii*

In intensive care units, *Acinetobacter baumannii* is a significant cause of nosocomial infections (e.g., pneumonia, bacteremia, wound infections) [94]. The appearance of multidrug-resistant strains has allocated this bacterium on top of the World Health Organization (WHO) list of pathogens for which new therapeutics are urgently needed [95].

Taking into account the use of ECM proteins as docking sites, it can be speculated that, in tissue damage circumstances, the exposure of ECM proteins might favor adherence and biofilm formation, complicating *A. baumannii* infection treatment [40]. *A. baumannii* has shown affinity to collagen and Fn [96], but only recently, the virulence factors and mechanism of the disease have been described in more detail [97].

*Acinetobacter* trimeric autotransporter (Ata) is a surface adhesin crucial for biofilm formation and binding to ECM components (collagen types I, III, IV, and V and Ln), but not to Fn and collagen type II [40]. Deletion of *ata* significantly diminished the binding of *A. baumannii* to endothelial cells in static and dynamic conditions, thereby highlighting the importance of this adhesin in the persistence of infection [98]. Additionally, Ata proved to be a potential vaccine candidate against *A. baumannii* as it was observed that the application of Ata-specific antisera attenuated the course of infection in mice [41]. It is worth mentioning that *ata* was present in 78% of the sequenced *A. baumannii* isolates but only in 3% of the closely related but much less human pathogenic *A. calcoaceticus/A. pittii* clade [99].

The impact of outer membrane proteins (OMPs) in the adhesion to Fn has been widely described [42]. The interaction occurring between OMPs and Fn might represent a critical step for lung epithelial colonization in *A. baumannii* mediated infections [97]. OmpA (previously known as Omp38) is a highly conserved OMP in clinical isolates and is one of the best-characterized virulence factors of *A. baumannii* [100–102]. Expression of *ompA* is associated with the cytotoxicity to eukaryotic cells and the adhesion to Fn [42, 103]. The interference of OmpA function by the pretreatment of *A. baumannii* with a binding-inhibiting synthetic hexapeptide resulted in a reduced bacterial adherence to lung human cells and Fn [104]. Moreover, Omp33 (also known as Omp33-36) is also involved in adhesion via Fn binding and invasion of human lung epithelial cells [42, 43, 105]. Finally, the TonB-dependent copper receptor (TonB), which facilitates the active transport of substances to the outer membrane, has also been described as a FnBP [42, 106].

### *Bartonella spp.*

The genus *Bartonella* compromises at least three species of major medical interest: *B. henselae* (cat-scratch disease), *B. quintana* (relapsing fever), and *B. bacilliformis* (Carrion’s disease). The bacterial transmission to humans occurs after contact with infected animals or via blood-sucking arthropods (vectors) [107]. *Bartonella* species are facultative intracellular bacteria with the capacity to colonize a wide broad of host cells, among them erythrocytes [108, 109], endothelial cells [110–113], monocytes, macrophages, and dendritical cells [114–116]. *B. quintana* and *B. henselae* have been described as endocarditis-causing agents [117–119]. The mechanisms occurring in infective endocarditis point to multifactorial events of bacterial adherence in which the interaction between bacterial adhesins and ECM proteins might fulfill a critical role even under pulsating and high shear stress conditions [98, 120, 121].

*Bartonella* adhesin A (BadA) is the representative adhesin of the species and was first described as a “type IV-like pilus” expressed by *B. henselae* [111]. Later research described the OMP as a TAA mediating bacterial binding to ECM proteins [44]. It was demonstrated that the presence of BadA in low passage bacteria facilitates adhesion and invasion in human epithelial cells [111]. After the utilization of wild-type strains and isogenic mutants, the mechanism of adhesion was found to be mediated by binding of BadA to ECM components (collagen III, Ln and Fn) under static and dynamic infection conditions [44, 98, 121]. Expression of truncated BadA-constructs (with deletions of specific subdomains) identified that the neck-stalk module of BadA is crucial for Fn-binding. This suggests that this neck-stalk module might mediate host cell adherence via interactions with, e.g., beta-1 integrins [45]. The head and a short part of the neck-stalk BadA module was found to be sufficient for collagen binding after expression of a truncated BadA hybrid in *E. coli* resulting in significantly higher adherence rates to endothelial cells than in *E. coli* controls [122]. These and other data demonstrate that the BadA protein is a major pathogenicity factor of *B. henselae* [44] and that the head and stalk domains of the protein have overlapping functions in the adhesion process [45].

Other bacterial surface proteins from *B. henselae* also show affinity to Fn. Pap31, a protein possibly involved in packaging or phage particle assembly, was described to be responsible for binding to immobilized Fn, specifically to the FnIII_12–13_ repeat module and to human umbilical vein endothelial cells (HUVECs) [46]. Additionally, Omp43, a porin protein [123], and Omp89 were identified as FnBPs after batch affinity-purification from OMPs binding to Fn-coated wells [47].

Among the genus, there are other important TAAs detectable: Variably expressed outer membrane proteins (Vomps) are a group of proteins identified as crucial for the pathogenicity of *B. quintana* as shown in a macaque animal infection model [48]. Amidst the four Vomps A to D proteins, VompA-C contains collagen-binding motifs, but only VompA and VompC showed collagen IV binding in in vitro assays [48]. The importance of Vomps in the adherence process was confirmed by binding experiments using in vitro cell culture vials and in dynamic experiments using capillary flow chambers, where the interaction between *B. quintana* and HUVEC was diminished in the absence of Vomps [121]. Due to the similarities shown between the BadA and Vomps, it was initially thought that Vomps were FnBPs; nevertheless, binding assays between Vomp and Fn did not show affinity, and this exemplifies the complexity involved in the prediction of binding interaction [112].

In the case of *B. bacilliformis,* little research has been done regarding adhesins and their interaction with ECM proteins. The TAA *Bartonella* repeat protein (Brp), also called *B. bacilliformis* adhesin A (BbadA), shares common domains and structural features with the already identified TAAs, BadA, and Vomps. For this reason, it is speculated that Brp/BbadA might be involved in similar biological processes including adhesion to host cells and ECM proteins [49, 124]. Additionally, hemin-binding proteins (Hbps) from *B. bacilliformis* are homologous to Pap31 from *B. henselae*. Some possible interaction of Hbps with Fn might be speculated because of this reported similarity [49]. The role of flagellin in Fn-binding of *B. bacilliformis* has not been analyzed so far.

### *Borrelia burgdorferi*

*Borrelia burgdorferi* is the causative agent of Lyme disease, one of the most common tick-borne diseases occurring in the Northern hemisphere. *B. burgdorferi* is a Gram-negative obligate extracellular spirochete, frequently found to be associated with its hosts’ connective tissues [53]. The persistence of spirochetes in joints and connective tissue is essential for the incidence and severity of the infection [125]. Bacterial adhesion to host tissue is, therefore, a critical step for the initial process of infection and the capacity of spirochetes to disseminate to distant organs.

*B. burgdorferi* expresses at least 19 OMPs, many of which are known to bind to host cells and ECM components [126, 127]. The BBK32 protein is a surface-exposed molecule first identified by its property to bind Fn [50]. Isogenic *bbk32*-deficient mutants showed an impaired ability to bind immobilized Fn and an attenuated adhesion to mouse fibroblast cells when compared to wild-type strains. Moreover, in a murine model of Lyme disease, mice infected with wild-type and isogenic mutants indicated a decreased infectivity of the *bbk32-*deficient strains highlighting the importance of BBK32 for initial infection [128]. Recent research revealed a 70-kDa N-terminal Fn region as the responsible element for BBK32 binding [129]. Regardless of the proven importance of the BBK32 protein in the infection process, the isogenic *bbk32*-deficient mutants are still able to bind Fn with a reduced capacity suggesting that additional mechanisms for Fn-binding exist. RevA, another borrelial outer surface protein was discovered to bind to the N-terminal Fn region in a comparable affinity as BBK32 based on their *K*_D_ values. RevA was also found to interact with Ln but in a lower affinity than BBK32 [57]. The RevA paralogous protein RevB was also identified as a FnBP after the evaluation of recombinant RevB protein in binding assays with Fn [57].

Spirochetes are often associated with connective tissues and collagen fibers in the infected mammalian hosts, suggesting the presence of adhesins for binding certain types of collagen. BBA33, a surface-exposed lipoprotein linked to bacterial virulence, has been proven to stick in high affinity to collagen type VI and to collagen type IV [52]. The affinity of *Borrelia* spp. to collagen fibers might also be related to the presence of collagen-associated proteoglycans such as decorin which is an abundant molecule in connective tissues. For instance, the decorin-binding proteins DbpA and DbpB display a strong affinity to decorin but lack binding affinity to collagen [130–133].

The first Ln-binding protein identified in *B. burgdorferi* was the OspE/F-related protein ErpX [55]. The interaction of this lipoprotein with Ln-containing host tissues favors migration through extracellular matrices with long-term colonization [56]. *Borrelia* membrane protein A (BmpA) is another LnBP. Binding of Ln with BmpA was obstructed upon the incubation of Ln with solubilized collagen. These competition assays localized the Ln-BmpA binding site at the collagen-binding region in Ln [53]. The BmpA paralogous proteins BmpB, BmpC, and BmpD have also been described as LnBPs [53]; all four paralogs are expressed during mammalian infections [134]. Of note, BmpA and BmpB are selectively expressed in joint tissues and, thus are involved in the genesis of Lyme arthritis [135].

Other borrelial adhesins comprise proteins displaying multi-functional properties; this is the case of complement regulator-acquiring surface protein 1 (CspA) and 2 (CspZ). Both proteins do not only bind to Fn and Ln but also to collagen type I, III, and IV with a stronger interaction in the case of CspA [54].

### *Campylobacter jejuni*

*Campylobacter jejuni* is recognized as a common cause for bacterial gastroenteritis. Like other intestinal pathogens, the capacity to colonize the gastrointestinal tract by binding epithelial cells is a fundamental step during the initial phases of infection.

CadF from *C. jejuni* is an OMP described for the first time in 1997 after the observation of reduced binding to Fn in an isogenic strain lacking CadF expression [59]. Later research applying overlapping peptides of CadF identified a four-amino-acid sequence responsible for Fn-binding. Modifications in this sequence resulted in a recombinant CadF protein that significantly showed reduced Fn-binding and adherence to epithelial cells compared to the wild-type [60]. As a proof of concept, reduction in the internalization of *C. jejuni* into human intestinal epithelial cells was observed in a CadF mutant strain [136]. Other possible functions of CadF still remain unclear; post-translational proteolytic cleavages of the protein in clinical *C. jejuni* revealed that small fragments of CadF still bind Fn and are no longer recognized by the host humoral response [137].

The recently detected FnBP from *C. jejuni* is encoded by the gene Cj1279c; the protein was termed as Fn-like protein A (FlpA) because of the presence of three Fn type III-like repeats in its protein structure [63]. Due to the description of Fn–Fn interactions located in Fn type III domains [18], it was hypothesized that FlapA might be involved in Fn-binding activity [63]. FlapA was found to mediate bacterial attachment to host epithelial cells via Fn-binding [61], and this interaction was described to occur through the Fn gelatin-binding domain and the second Fn type III-like repeat from FlpA [62]. CadF and FlpA have probed to act together in cellular membrane rearrangements and epithelial cells invasion [138].

Other adhesins have also been identified in *C. jejuni*: for instance, Cj1349c has been annotated as a putative Fn/fibrinogen-binding protein because of observed reduced binding in isogenic forms of the protein in vitro; however, its functional role in vivo is still unknown [63]. Additionally, adhesion of *C. jejuni* to collagen and Ln (only under high concentrations) has been reported via in vitro binding experiments using coated coverslips and OMP suspensions [139, 140].

### *Escherichia coli*

*Escherichia coli* has been associated with diarrheal illness ranging from acute to long-lasting stages in developing and industrialized regions of the world. The worldwide spread of the bacteria has promoted the need to unravel the pathogenic mechanisms applied by this pathogen to colonize and infect intestinal cells. Several adhesins have been recognized for binding ECM proteins that are naturally present in epithelial cells; among them are flagella, aggregative adherence fimbriae (AaF), long-polar fimbriae (Lpf1), curli, and UpaB, highlighted for their multiple binding specificities.

The flagella of enteropathogenic and enterohemorrhagic *E. coli* contribute to host-colonization. Flagellin of enteropathogenic *E. coli* binds in a dose-dependent manner to collagen and to a lesser extent to Ln and Fn [64]. A more recent report demonstrated higher affinity of flagellin from an atypical enteropathogenic *E. coli* to cellular Fn, underlining the high variability of virulence strategies among this species [65]. Oppositely, flagellin of enterohemorrhagic *E. coli* has almost no selectivity for ECM proteins [64]; therefore, its contribution to host colonization might be related to other mechanisms.

In enteroaggregative *E. coli*, the AaF II protein contributes to the adherence to human intestinal tissue. Farfan et al. reported that enteroaggregative *E. coli* adhered more abundantly to surfaces precoated with Fn, Ln, and type IV collagen than a strain with a mutation in the AaF II major pilin gene, concluding that Fn-AaF II binding may contribute to colonization of the gastrointestinal tract [66]. Further research focused on the participation of α5β1 integrins in the Fn-mediated adherence of AaF II to intestinal cells. It was shown that enteroaggregative *E. coli* binds indirectly to integrin α5β1 (mediated by AaF II and Fn interaction), but remarkably it can also bind directly to integrin α5β1, presumably by the interaction of another so far uncharacterized adhesin [141].

Lpf1 protein from enterohemorrhagic *E. coli* has been associated with increased adherence to cultured cells (Caco-2, HeLa-229) [142, 143]. In line with that observation, it was shown that mutations in the LP fimbrial operons of the *lpf* genes lead to a decreased colonization in animal models [144]. The adhesive properties of Lpf1 were described to be mediated via ECM protein binding, as inactivation of the *lpfA1* gene significantly reduced the binding of *E. coli* mutants to Fn, Ln, and collagen IV [69].

Curli are thin surface fibers expressed by many pathogenic isolates of *E. coli* and other bacteria associated with severe infections in humans. Among clinical isolates of *E. coli*, most enterohemorrhagic, enterotoxigenic, and sepsis-related strains express curli, in contrast to enteroinvasive and enteropathogenic strains, which lack curli expression. This difference in expression suggests a versatile role of curli fibers in pathogenicity [67, 145]. Curli fibers have been described as adhesins for their binding capacity to host molecules such as Fn and Ln. Remarkably, their ability to bind Fn has demonstrated to be an important factor for the internalization of bacteria in eukaryotic cells [145]. Recently, using a nanomechanical force-sensing approach, it was identified that curli and Fn formed multiple specific bonds with high tensile strength, resulting in tight *E. coli* binding [68].

UpaB, an autotransporter of uropathogenic *E. coli* strains, is known to contribute to the colonization of the urinary tract and promotion of bacterial binding to the ECM proteins Fn, fibrinogen, and Ln, but not collagen (type I, II, III, IV, and V) [71]. Due to the observation of stronger affinity between UpaB and Fn, the molecular interaction between these two proteins was analyzed at a molecular level. Ten residues in UpaB (D116, D119, N146, N175, D217, K245, D246, D281, R310, and D336) demonstrated to be necessary to maintain the secondary structure of UpaB and to mediate Fn binding; from the Fn site, binding of UpaB was located at the FnIII region, most likely at the FnIII_1–2_ [70]. Summarizing, the interaction between the two proteins involves the folding of a β-helix in UpaB presenting charged/polar residues which interact with charges on the FnIII domain [70].

### *Haemophilus influenzae*

*Haemophilus influenzae* is often found as a commensal of the respiratory tract but also represents a common cause of respiratory tract infections and meningitis. The presence of a polysaccharide capsule classifies *H. influenzae* in encapsulated strains responsible for invasive disease and unencapsulated (nontypeable NTHi) strains found in mucosal infections in the upper and lower respiratory tract [146]. *H. influenzae* prefers binding to non-ciliated cells, areas with damaged epithelium and mucus present in the respiratory tract [147] via a number of OMPs that influence the process of adherence and colonization [148].

The *Haemophilus* adherence and penetration protein (Hap) is a classical autotransporter adhesin ubiquitously present among *H. influenzae* type b encapsulated and NTHi clinical strains. Hap promotes bacterial adherence to epithelial cells and mediates bacterial aggregation and microcolony formation by Hap–Hap interactions occurring between neighboring bacteria [73]. The passenger domain of the protein harbors a serine protease activity that directs autoproteolytic cleavage under dispersal and migration from the site of infection [147]. The mutation on the serine active site inhibits the release of Hap from the bacterial surface and results in increased adherence to epithelial cells [73]. Hap binds to Fn, collagen IV (but not collagen II), and Ln. Inhibition of bacterial binding to ECM proteins after the application of polyclonal antiserum against the passenger domain confirmed the importance of Hap in the infection process [72]. Hap was found to interact with the FnIII_1–2_ region, a domain in Fn previously described as crucial for matrix assembly [18]. The interaction between Hap and FnIII_1–2_ might indicate that Hap is involved in the destabilization of the Fn matrix enabling the spread of *H. influenzae* through the submucosa to the basement membrane [75].

The surface lipoprotein *Haemophilus* protein E (PE) is a highly conserved protein among the *Haemophilus* spp. members [149]. PE induces the pro-inflammatory response during infection and promotes bacterial adherence and invasion through the binding of the N-terminal PE and the Ln globular domains [76, 79], which also happens with a simultaneous interaction of PE with vitronectin [78]. Moreover, *Haemophilus* protein F (PF), a ubiquitous protein of *H. influenzae*, was described as a LnBP after the observation of reduced Ln-binding and human pulmonary epithelial cells’ attachment in an isogenic *hpf* mutant [150].

The observation that *H. influenzae* mutants lacking expression of the known ECM-binding proteins (Hap, PE and PF) still bind ECM proteins aroused the interest in other bacterial adhesins. To further study this, OMPs of *H. influenzae* were analyzed in regard to their ECM interactions. A 28-kDa protein, later identified as *Haemophilus* lipoprotein e (P4), was found binding to Ln, Fn, and vitronectin by a strong interaction (constant dissociation Kd: 9.26 nM, and 10.19 nM and 16.51 nM, respectively) [80]. P4 is present in NTHi and encapsulated *H. influenzae* and was previously described as important for NAD uptake and hemin utilization [151, 152]. Interactome studies of Ln and NTHi strains gave a major description of interactions occurring between already known and novel LnBPs [76].

Although all adhesins (Hap, PE, PF and P4) exhibit binding capacities to ECM proteins, Hap has shown the highest binding capacity to Fn. In contrast, Ln binds almost equally well to all the bacterial adhesins [80]. It seems that not a single OMP of *H. influenzae* is responsible for bacterial adhesion to the host but a coordinated interaction between adhesins and host proteins.

### *Yersinia spp.*

The genus *Yersinia* harbors 17 different species but only three of them have been described to be pathogenic to humans (*Y. enterocolitica*, *Y. pseudotuberculosis, Y. pestis*). *Y. enterocolitica* and *Y. pseudotuberculosis* are causative agents for a wide range of diseases associated with the consumption of contaminated food. *Y. pestis* is a zoonotic pathogen transmitted by fleas from one mammalian host to another. Three major adhesins have been described: the *Yersinia* adhesin A (YadA), invasin (Inv), and attachment invasion locus (Ail). These adhesins mediate attachment to host cells, either directly via binding integrins (as for invasin) [153, 154] or indirectly via ECM proteins. This attachment is a crucial prerequisite for injection of effector proteins (*Yersinia* outer proteins, Yops) via a type three secretion system and thus essential for a successful host cell invasion. Both YadA and Ail have been shown to bind Fn and Ln proteins [91], whereas only YadA may interact with collagen [155, 156].

YadA is by far the best described TAA. This protein is essential for virulence in *Y. enterocolitica* and is encoded by the pYV virulence plasmid. In contrast to *Y. enterocolitica*, *Y. pseudotuberculosis* does not require YadA for a successful colonization process [157, 158] and *Y. pestis* does not express the *yadA* gene due to a frameshift caused by a nucleotide deletion [159–161]. Among the manifold virulence traits associated with YadA, one very important role is the mediation of bacterial adhesion. YadA of *Y. enterocolitica* stably interacts with collagen types I, II, III, IV, V, and XI [155]. The YadA-collagen type I interaction has been studied in most detail due to the strong interactions. It was discovered that the trimeric form of the YadA head domain is essential for binding [90]. However, a specific sequence responsible for the interaction with collagen was not identified so far. Therefore, it is assumed that the binding motif is formed by a specific fold instead of a distinct peptide sequence [89]. Compared to most serotypes of *Y. enterocolitica*, the YadA of *Y. pseudotuberculosis* possesses an additional short stretch within its head domain. This region significantly determines the differential ECM-binding repertoire of the two *Yersinia* species. Actually, the deletion of this region abrogated Fn-binding but increased binding to collagen by four- to fivefold and significantly enhanced binding to Ln [158]. YadA of *Y. enterocolitica* also binds Fn and Ln, however, with a significantly lower affinity compared to collagen and using a different binding region in the YadA molecule [121, 156]. *Y. enterocolitica* strains of serotype O:9 contain a similar stretch and it was shown that this stretch is responsible for vitronectin binding [162]. The YadA–collagen interaction can even resist harsh conditions as incubation at 80 °C for 20 min, pH values from 5.0 to 10.0, proteolytic treatment, and incubation in 1 M urea [163].

Ail is a 17-kDa chromosomally encoded protein also associated with *Yersinia* virulence. Ail is present in *Y. pestis*, *Y. enterocolitica,* and *Y. pseudotuberculosis,* but the similarity between the protein sequences in the species is rather low [164]. Ail accomplishes an important activity in binding to ECM proteins (especially to Ln and Fn) with no detectable binding to collagen [91, 92]. Contrarily, in *Y. pseudotuberculosis* Ail lacks the conserved residues responsible for binding [165] but still accomplishes functions related to serum resistance. As Ail is a small-sized protein, its functions may be masked by LPS outer core and/or O-antigen and this has been shown already for *Y. enterocolitica* and *Y. pseudotuberculosis*. Therefore, Ail may only contribute to adhesion in strains expressing rough LPS, such as *Y. pestis* [166, 167].

## ECM interactions occurring in other Gram-negative bacteria

For some genera of Gram-negative bacteria, the interactions occurring between adhesins and Fn, Ln or collagen are not critical for bacterial attachment, but the description of bacterial-host receptor occurrence is vital for understanding the orchestrated process of infection.

*Salmonella enterica* serotype Typhimurium expresses adhesins such as ShdA, MisL, and SadA, of which the last one is a TAA. ShdA and MisL are type Va autotransporter adhesins that have proved to contribute to intestinal colonization possibly by binding to Fn. The interaction of ShdA and Fn happens between the passenger domain and the FnIII_13_ module, respectively [84, 85], while the interaction occurring between MisL and Fn has not been further described [87, 168]. Even though SadA shares structural similarities with YadA and BadA, current studies did not succeed in identifying any SadA-mediated interactions with ECM molecules. However, it was shown that SadA plays a role in biofilm formation and adhesion to human intestinal epithelial cells via a yet unknown mechanism [169]. Additionally, Rck and PagC, *Salmonella*-homologs of Ail of *Y. pestis*, have proved to induce bacterial binding to Ln and Fn, when these adhesins are expressed in *E. coli* [88, 91].

*Pseudomonas aeruginosa* is an opportunistic pathogen affecting mainly immunocompromised patients and also has demonstrated affinity to ECM proteins (Fn and Ln). The outer membrane porin Q (OprQ), known to play an important role in membrane permeability, antibiotic resistance, and virulence, was later identified as a FnBP. Based on the observation that the expression of Fn is positively correlated with the degree of injury in affected tissue from lung epithelial cells [170], it seems to be possible that overexpression of Fn favors the adherence and colonization of *P. aeruginosa* in patients promoted by OprQ [82]. Moreover, Ln is a ubiquitously expressed ECM protein in the respiratory tract and is a highly important target for Paf (orthologue protein of *H. influenzae* protein F) in *P. aeruginosa* also facilitating bacterial adherence [83].

*Helicobacter pylori* is the etiological agent of gastritis and malignant neoplasias, such as gastric cancer. The adherence to the gastric epithelium has been shown to enhance inflammation, yet only a few *H. pylori* adhesins have been paired with targets in host tissue. *H. pylori* was described as binding some ECM proteins with different affinity: vitronectin [171], collagen type IV, and Ln [172]. The binding capacity of *H. pylori* to collagen type IV and Ln was reduced when the bacteria was subjected to proteolytic enzymes, suggesting that the bacterial attachment to the basement membrane is mediated by bacterial surface proteins [172]. Later research demonstrated that *H. pylori* mutants lacking the adhesins AlpA and AlpB showed reduced binding to Ln, while expression of a plasmid containing the alpAB locus in *E. coli* conferred Ln-binding capacity. Surprisingly, such mutants did not show lesser inflammation capacity than the wild-type when gerbils were experimentally infected [81].

## Other interactions related to ECM binding proteins: degradation of ECM proteins by bacterial proteases

In order to reach the host tissue, bacteria can make use of endogenous proteases to degrade the basement barrier protecting the ECM [4]. Presence of proteases has been more commonly described for fungi and parasites. However, the activity of a few bacterial proteases in the host ECM has also been observed. Examples are elastase and alkaline proteases produced by, e.g., *P. aeruginosa*; both enzymes target soluble Ln resulting in different cleavage products [173]. Another example is a chymotrypsin-like protease from *Treponema denticola* which allows bacterial invasion of the basement membrane after degradation of Ln, collagen IV, and fibrinogen [4, 174]. In *H. pylori*, proteases of the high-temperature requirement A (HtrA) family have ECM protein degradation activity. HtrA proteins cleave Fn in in vitro assays, suggesting implications of the secreted HtrA in the infection process and in the disruption of the epithelial barrier [175]. In *Y. pseudotuberculosis*, the Pla protein has protease activity degrading Ln and fibrin during the invasion of epithelial cells [176].

## Inhibition of bacterial adhesion as a possible therapeutic strategy

The overwhelming increment of antibiotic resistance in many clinical bacterial isolates has stimulated the scientific and medical interest for the development of new approaches directed to combat serious infections. Many strategies designed against bacterial survival and growth pathways are already established; nevertheless, the selective pressure imposed by bactericidal promotes continuously the spread of resistant strains among clinical patients. This fact underlines the need for redirecting the focus to alternative therapeutic targets. Furthermore, the ambitious idea of blocking virulence factors associated with bacterial colonization and infection processes is an attractive strategy to possibly prevent infections, to attenuate already existing infections, and to promote the natural clearance of the pathogen [3, 29, 39, 177].

As stated before, adhesion plays a primordial role at distinct steps of the infection process; therefore, the attempt of targeting this interaction by the application of “anti-adhesion”-therapeutics is not new and has been approached previously. One example is the type I pili adhesin of uropathogenic *E. coli* (FimH), which binds to mannosylated receptors on the surfaces of mammalian bladder epithelial cells. Anti-adhesion agents targeting FimH-mannosylated interaction have been observed either disrupting the adhesin protein directly [178, 179] or interfering with the binding by fitting the binding pocket of the host receptor in FimH (FimH antagonists) [180–182]. In murine models, administration of a FimH antagonist has proven to decrease bacterial colonization to similar levels as by antibiotic treatment suggesting an attractive alternative to classical antibiotics [183, 184]. However, it is worth mentioning that no antivirulence agent against uropathogenic *E. coli* has been tested in humans so far.

Alternatively, the observation of mucus secretion as a natural defense mechanism against enteropathogens guided the scientific interest in studies of a variety of mucin glycoproteins mimicking the glycosylation patterns present in epithelial surface receptors. Mucins bind and immobilize bacteria, favoring the bacterial clearance by discharging the mucus layer from the gastrointestinal tract [185]. Purified bovine mucin (Muc1) extracted from cow milk inhibited the binding in vitro of Gram-negative pathogens (*E. coli* and *S. enterica* serovar Typhimurium) to intestinal epithelial cells [186].

Peptide-based adhesion inhibitors represent another attractive approach to interfere with bacterial adherence. The feasibility of large-scale production and effectiveness in in vitro assays makes them an attractive target for an antiadhesive therapy approach. A promising example is represented by the multivalent adhesion molecule (MAM7), a bacterial surface protein involved in the attachment of a range of Gram-negative bacteria (enteropathogenic *E. coli*, *Y. pseudotuberculosis* and *Vibrio* spp.) to the host cell membrane receptors (Fn, phosphatidic acid) [187, 188]. An in vitro assay using bead-coupled MAM7 successfully reduced the cytotoxicity of host cells. It should be noted that this approach was not interfering with the bacterial-host receptor binding process directly, but via a bacterial-host receptor competition strategy after the pre-exposition of the host-receptor binding pocket to bead-coupled MAM7. The results brought to light a potential application of MAM7 as a prophylactic agent against multidrug-resistant bacterial pathogens [189, 190].

Moreover, elucidating the molecular mechanisms responsible for the anti-adhesive capacity of bioactive natural compounds from plants might give some insights about strategies to block bacteria–host interactions avoiding endogenous impact on cellular host signaling. Some examples are the anti-adhesion activity described for salvianolic acid B (SA-B) against *Neisseria meningitidis* [191], the use of cranberry proanthocyanidins against uropathogenic *E. coli* [192, 193], and the identification of anti-adhesive peptides against a *H. pylori* FnBP obtained by the enzymatic hydrolysis of pea seeds *Pisum sativum* [194]. The presence of food components contrasting bacterial adhesion has been reviewed extensively [195].

Despite the promising future of anti-adhesion therapy, several considerations have to be made before the application of this concept to patients. First, the redundancy of the interaction between adhesins and cellular receptors represents a challenge. For instance, TAAs such as BadA and YadA show affinity to various ECM proteins [44, 156], while curli and flagellin from *E. coli* strains bind not only ECM proteins but also cellular receptors involved in immunity [196, 197]. Moreover, many pathogens such as *P. aeruginosa* express a wealth of adhesion and other virulence factors that may act in concert and/or redundantly. Blocking the function of a single adhesion molecule would, therefore, be an unsuccessful therapeutic strategy. Additionally, the design of high-affinity anti-ligands might be considered as a milestone for the treatment of bacterial infections. The similarity between pathogenic adhesins and extracellular protein domains, as the case of FlpA from *C. jejuni* and Fn type III domains [63], implies that interfering with pathogen attachment might compete with host signaling pathways leading to undesirable consequences [198]. Another drawback is the fact that adhesins are often expressed only at very distinct time points of the infection process, as shown for YadA and invasin of *Y. enterocolitica* [156, 199, 200]. Based on this, the successful application of anti-ligands for treatment (once they have been designed) will depend strongly on the time point when these compounds are administered to a patient.

In summary, multiple strategies for bacterial attachment to the host exist and they are highly regulated and orchestrated during the entire course of an infection [201]. Attempts to overcome the limitations of an anti-adhesin therapeutic strategy will thus require also a time-resolved and tissue-specific understanding of the host cell signaling events occurring during the infection process. Even so, and in the light of the global threat of emerging and spreading antibiotic-resistant pathogens, anti-adhesion and in a more general way anti-virulence therapies might be a worthwhile alternative to classical antibiotic treatment.

## Concluding remarks

As we gain more and more insights into the interactions that occur during host cell adhesion, colonization, and the invasion processes of pathogenic bacteria, the complexity of these interactions becomes obvious. The evolution of adhesin structures and the redundancy for cellular targets suggest a dynamic interaction and adaptability to the particular conditions in host tissues. This creates a challenge for researchers aiming at the inhibition of host–pathogen interactions. Even though matrix-binding proteins have been studied for more than 40 years, it is still a long way to comprehensively understand the underlying molecular mechanisms important for the bacterium–host interplay. Because of the current limitations associated with the application of any anti-adhesion therapy, further efforts are necessary to better understand these interactions in the search for therapeutic alternatives to overcome the severe threat by multi-drug resistant Gram-negative bacteria.
